# Performance Analysis of Anode-Supported Solid Oxide Fuel Cells: A Machine Learning Approach

**DOI:** 10.3390/ma15217760

**Published:** 2022-11-03

**Authors:** Mohammad Hossein Golbabaei, Mohammadreza Saeidi Varnoosfaderani, Arsalan Zare, Hirad Salari, Farshid Hemmati, Hamid Abdoli, Bejan Hamawandi

**Affiliations:** 1 School of Metallurgy and Materials, College of Engineering, University of Tehran, Tehran 1417935840, Iran; 2 School of Metallurgy and Materials Engineering, Iran University of Science and Technology, Tehran 1684613114, Iran; 3 Renewable Energy Research Department, Niroo Research Institute (NRI), Tehran 1468613113, Iran; 4 Department of Applied Physics, KTH Royal Institute of Technology, SE-106 91 Stockholm, Sweden

**Keywords:** solid oxide fuel cell (SOFC), machine learning, neural network, gaussian process, SOFC performance

## Abstract

Prior to the long-term utilization of solid oxide fuel cell (SOFC), one of the most remarkable electrochemical energy conversion devices, a variety of difficult experimental validation procedures is required, so it would be time-consuming and steep to predict the applicability of these devices in the future. For numerous years, extensive efforts have been made to develop mathematical models to predict the effects of various characteristics of solid oxide fuel cells (SOFCs) components on their performance (e.g., voltage). Taking advantage of the machine learning (ML) method, however, some issues caused by assumptions and calculation costs in mathematical modeling could be alleviated. This paper presents a machine learning approach to predict the anode-supported SOFCs performance as one of the most promising types of SOFCs based on architectural and operational variables. Accordingly, a dataset was collected from a study about the effects of cell parameters on the output voltage of a Ni-YSZ anode-supported cell. Convolutional machine learning models and multilayer perceptron neural networks were implemented to predict the current-voltage dependency. The resulting neural network model could properly predict, with more than 0.998 R^2^ score, a mean squared error of 9.6 × 10^−5^, and mean absolute error of 6 × 10^−3^ (V). Conventional models such as the Gaussian process as one of the most powerful models exhibits a prediction accuracy of 0.996 R^2^ score, 10^−4^ mean squared, and 6 × 10^−3^ (V) absolute error. The results showed that the built neural network could predict the effect of cell parameters on current-voltage dependency more accurately than previous mathematical and artificial neural network models. It is noteworthy that this procedure used in this study is general and can be easily applied to other materials datasets.

## 1. Introduction

Among different types of fuel cells, solid oxide fuel cells (SOFCs) result in higher efficiency in energy conversion than the others [1]. Numerous authors have carried out investigations to model and optimize the SOFC technology. Modeling methods, generally used to minimize the challenges of SOFC development, might need different types of equipment or long run times. These models are based on the mathematical relations demonstrating the physiochemical and electrochemical reactions taking place at/between different components of a single SOFC [2]. Moreover, developing an applicable mathematical model sometimes requires considering various variables related to the microstructure and electrochemical properties of the component-given material, SOFC working condition, and physiochemical mechanisms. Therefore, a complicated model should be solved with simplifying assumptions or complex numerical solutions. For example, a model was proposed to simulate SOFC performance at different pressures, temperatures, and gas flow by means of computation three-dimensional fluid dynamics (CFD) simulation [3]. Additionally, a three-dimensional model was proposed to investigate the maximum temperature and distribution at different working states [4]. Moreover, a two-dimensional thermos-fluid model for analyzing the effect of gas composition, flow rate, and porosity as a microstructural feature was introduced by coupling an electrochemical model with a CFD model. So, this model can describe transport and reaction phenomena in a SOFC [5].

Fuel cell performance is normally characterized by its polarization curve, which is a plot of its potential versus current density. The obtained data can be further used to predict the current-voltage dependency by Machine Learning (ML) models. These predictions are usually of higher accuracy than conventional modeling results, and the algorithms within the ML can establish authentic relationships based on empirical data. Supervised learning has increasingly attracted attentions in the field of material science and engineering thanks to its unique advantages compared with other mathematical and modeling approaches [6,7,8,9]. The major difference between ML and other mathematical modeling relies on the fact that statistical optimization requires established relationships among variables and some initial assumptions. In such a context, ML models could solely find the pattern and relation between all the data without any presumption [10]. The major goal of ML is to find a hypothetical function enabling scientists to predict new output values based on the proposed model. It can also save time, cost, experimental materials, etc. [11].

Traditionally, the commercialization process of the SOFC system includes several factors such as materials consumption, numerous experiments, and long-term stability testing. Considering these factors is time-consuming and requires a great amount of funding. Regarding hindering these limitations, some mathematical models and simulations were conducted, as was mentioned earlier. One of the approaches within which the mathematical models were utilized is the optimization of the cell parameters. Nevertheless, complexity of the SOFC system and calculations, simplifying assumptions, and the prerequisite knowledge over the physiochemical characteristics of a cell lead to insufficient accuracy of the predictive models [12]. Machine learning methods could be exploited to overcome the aforementioned limitations, and also a comparison of these methods’ prediction results would provide the best possible model with higher accuracy [13].

Considering the ML method as a new and proper modeling candidate, investigations have been focused on this area. Some researchers conduct ML methods for performance prediction, and some focus on comparing different models to enhance prediction results with less errors. In this regard, an artificial neural network (ANN) was conducted to predict the output voltage of cells in anode support SOFC, and some variables such as temperature, porosity, cathode layer thickness, electrolyte thickness, anode thickness, and current density were selected as input features. The current–voltage response was predicted under different circumstances, and the standard error of prediction of 1.705% was reported [14]. In another research, for a data set based on different parameters of Proton Exchange Membrane fuel cell (PEMFC) and its polarization behavior Support Vector Machine (SVM) and ANN techniques were utilized for comparing these methods and cell performance prediction [15]. Moreover, in another study on PEMFC, 9725 experimental data were collected from an electric bicycle powered by PEMFC, and these data sets were modeled by SVM and ANN. The predicted voltage–current, power–current, and efficiency power were achieved with 99% of accuracy for SVM and 97% for ANN [16]. Furthermore, support vector regression (SVR) and random forest (RF) algorithms were conducted to predict the performance of a SOFC cell, and the effects of hydrogen purity on the fuel mixture and temperature on cell output were investigated by ML. The results showed that the SVR algorithm depicts lower error values and better prediction [17]. Moreover, 858 data from 30 pieces of SOFC stack tested at four different operating conditions were utilized to predict stack performance using back propagation neural network (BP), SVM, and RF algorithms. These algorithms showed prediction errors of 1%, 4%, and 6%, respectively [18].

Various experiments, modeling, and data-driven methods have been applied and used to capture the relations between the cell parameters of SOFCs and current–voltage dependency [19,20,21,22]. Previous studies built some machine learning models and predicted target values or compared the models and described the best one [16,17,18]. In this study, a group of data related to the architectural and operational variables was used. Convolutional machine learning models and a multilayer perceptron (MLP) neural network were implemented to predict the current–voltage dependency as a function of the operational temperature, anode support thickness and porosity, electrolyte and cathode functional layer thickness, and compare the models’ efficiency to experimental results. Moreover, a comparison was performed between the obtained machine learning model and previous ones which employed neural networks and mathematically developed equations to justify the accuracy of predicted values from previous experimental results [14,23]. Then, the relations between cell design, operating temperature, and output voltage of a single cell of an SOFC were captured, and the performance was evaluated.

## 2. Dataset and Preprocessing

### 2.1. Machine Learning

**Figure 1:**
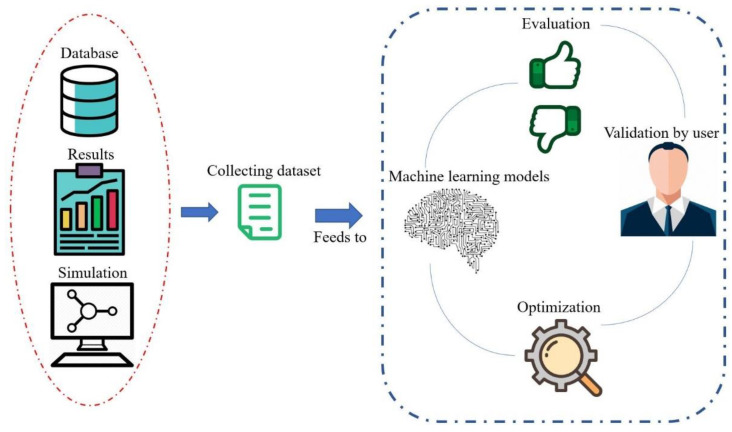
A general framework of the machine learning approach.

Machine learning as a data-driven approach could be adopted in the field of fuel cell engineering to predict, optimize, and modify the performance of fabricated cells. Three main steps are involved in this procedure: (i) collecting an appropriate dataset from a related database, experimental results, and simulation; (ii) data preprocessing, feature extraction and identification; (iii) building proper machine learning models, training, testing, and evaluating them on collected data. The framework of the mentioned procedure is illustrated in Figure 1.

### 2.2. Dataset 

The dataset used in this study was collected from an experimental work presented by Zhao and Virkar in 2005 [24]. The collected data contained 403 observations and six numeric features as fuel cells properties: working temperature, anode support layer (ASL) thickness, ASL porosity, electrolyte layer (EL) thickness, cathode functional layer (CFL) thickness, and the current density. Five disparate layers of these cells made from Ni/Yitria-stabilized Zirconia (YSZ) anode support, porous Ni/YSZ anode, dense YSZ electrolyte, porous Strontium doped Lanthanum Mangenite (LSM)/YSZ cathode, and porous LSM cathode current collector layers [24]. The dataset’s target value was the fuel cells’ output voltage. These data were chosen because the researchers evaluated the effect of the aforementioned parameters comprehensively, which made it favorable to categorize the data and satisfy all the required information for modeling. Moreover, there are some modeling in the literature on this dataset, which made us try to improve the built models. All codes were implemented by Python 3 using Scikit-learn, Seaborn, and Yellowbrick libraries.

### 2.3. Data Preprocessing

To apply the best machine learning models to the data, a standard preprocessing is essential to provide meaningful data for the statistical analysis and model fitting [25]. As the first step, data were divided into two groups: ‘X’ as input features and ‘y’ as the target value. Standard scaler was used for scaling the features to a desired range and form. Standard scaler not only transforms the features’ mean value to zero but also yields the features’ standard deviations to a unique calculated value and makes the features comparable and meaningful for the models.

The distribution of the data is essential for machine learning models. The correlations between the features also play an essential role in modeling. Principal component analysis (PCA) is mainly used for dimension reduction purposes. However, the PCA was also used to achieve good data distribution and minimize the correlation between the features [26]. In this study, PCA was used to minimize the correlations and modify the data distributions by mapping the features into specific new components. The new components not only have a minimum correlation coefficient, which is suitable for machine learning models, but also carry the same information for the model as features.

### 2.4. Splitting the Dataset to ‘X’ and ‘y’ and Standardization

The dataset attributes were first divided into two groups: ‘X’ and ‘y’. The ‘X’ group contained the experimental parameters (features) including temperature, ASL thickness, ASL porosity, EL thickness, CFL thickness, and current density. The ‘y’ group represents the target of the experiment, i.e., voltage. The standard scaler was used from the scikit-learn library in order to standardize the features.
from sklearn.preprocessing import StandardScalerX = data[[‘Temperature’, ‘AS thickness’, ‘AS porosity’, ‘Electrolyte thickness’, ‘CFLthickness’, ‘Current density’]]y = data[‘Voltage’]sc = StandardScaler()X_std = sc.fit_transform(X)

### 2.5. Minimizing the Features Relation and the Distributions Modifications

**Figure 2:**
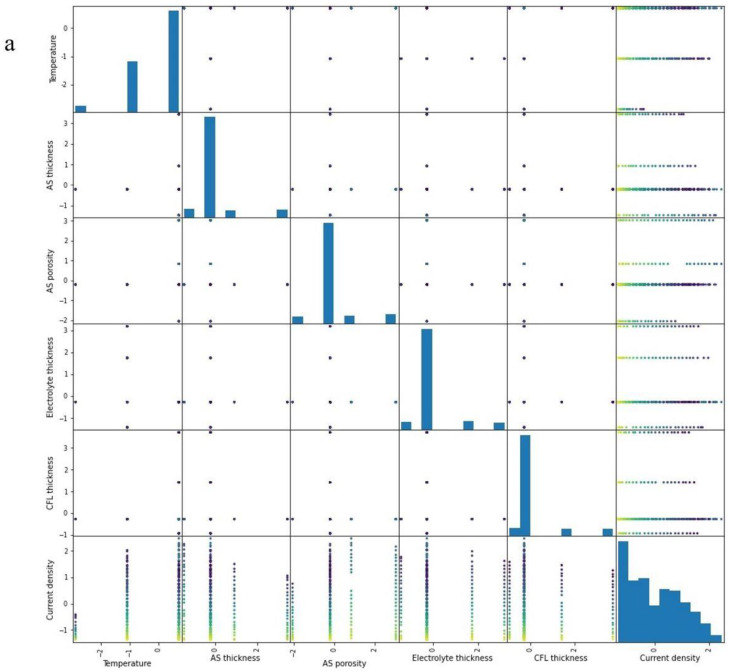

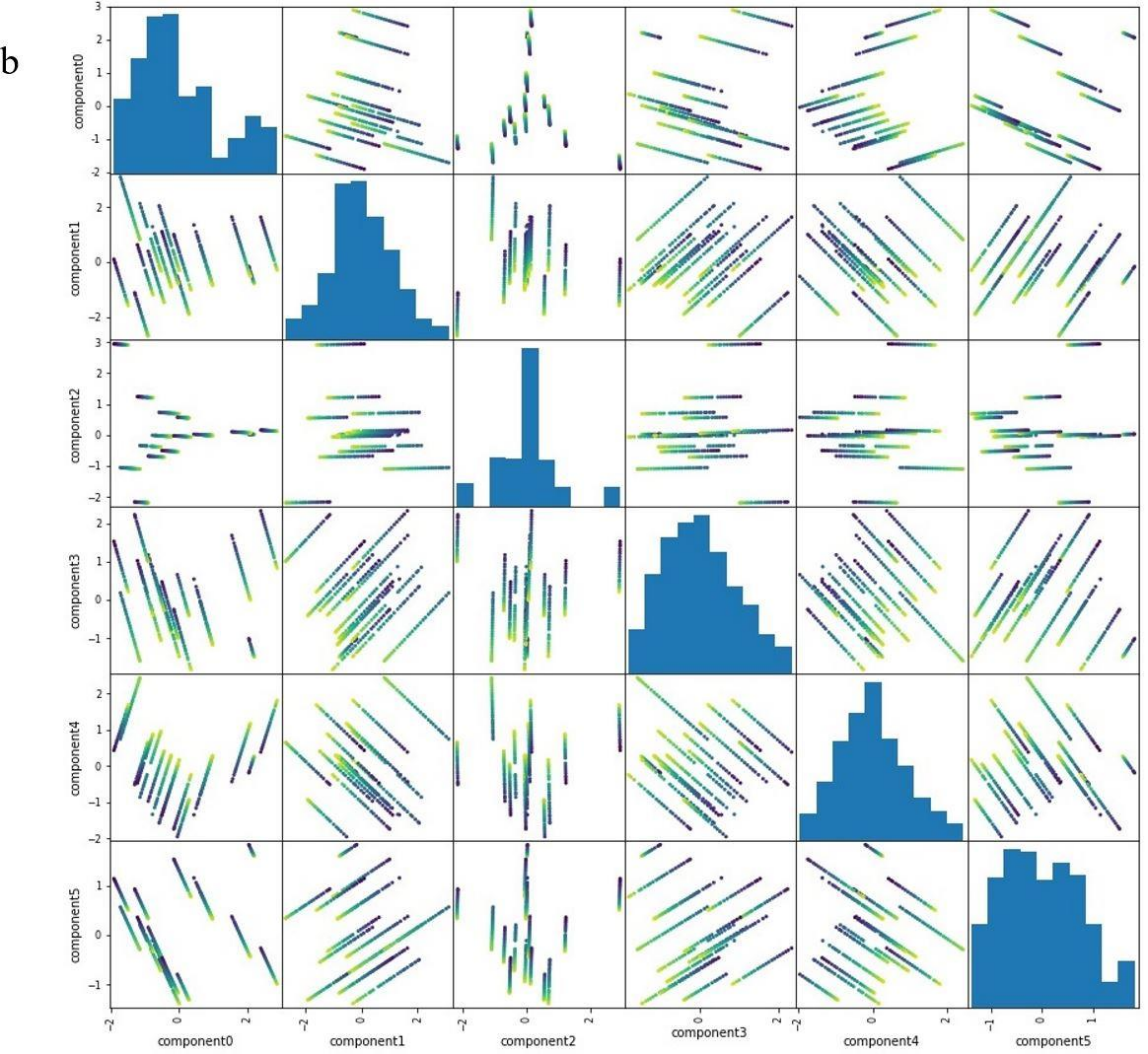
Distributions of (**a**) standard original features and (**b**) decomposed features using PCA.

Lowering the Pearson correlation of features for further processing stage and distributions modification, PCA was used from the scikit-learn library. The results of decomposition of features distribution is presented in Figure 2 by plotting using the pandas library with pandas.plotting.scatter_matrix. As described earlier, using PCA transforms the features into new components, which carry the same information for the model with a minimum correlation coefficient.

**Figure 3:**
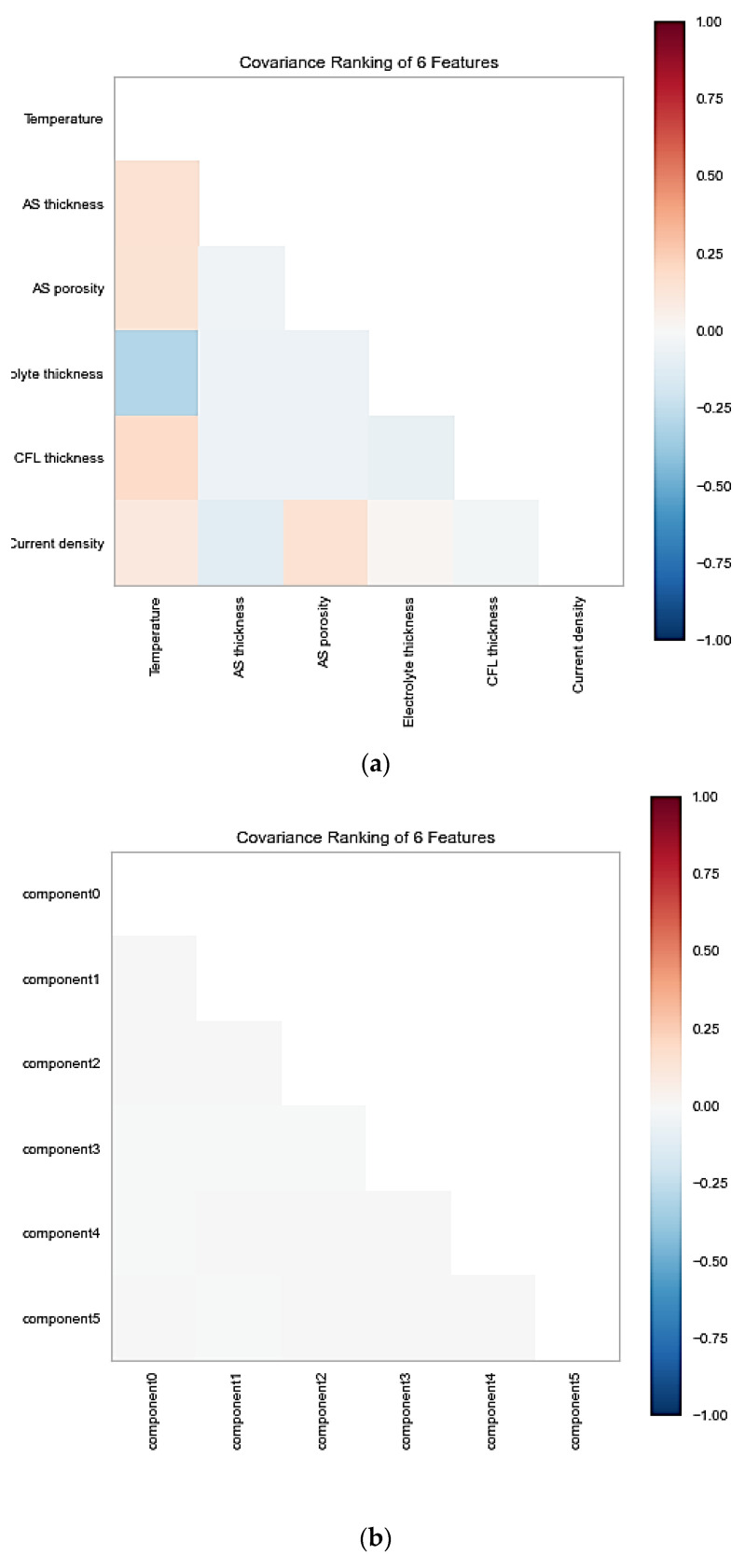
Covariance ranking of (**a**) standard original features and (**b**) decomposed features using PCA.

It is recommended that the low relations between the features could be more favorable for the models to learn the parameters between the variables because the highly correlated features (Pearson correlation value close to |1|) usually indicate similar information and linear trends between two features, which may lead to a decrease in the learning ability of the machine learning models [27]. To determine the correlations between the features, the covariance algorithm and rank2D were used from the yellow brick and matplotlib library, respectively, to visualize the covariance ranks matrix. The covariance ranks between features before and after using PCA are shown in Figure 3. All the features had covariance rank between −0.25 to 0.25, and there were no highly correlated features. However, the PCA was used to having some new components that are all independent because all the features in the performed experiment are independent. PCA also measures and reports the variance carried out by each new component.

The used codes for PCA to reduce the dimensions and correlations of the features mentioned below:
from sklearn.decomposition import PCApca = PCA(n_components = 6)X_decomposited = pca.fit_transform(X_std)

### 2.6. Feature Importance

**Figure 4:**
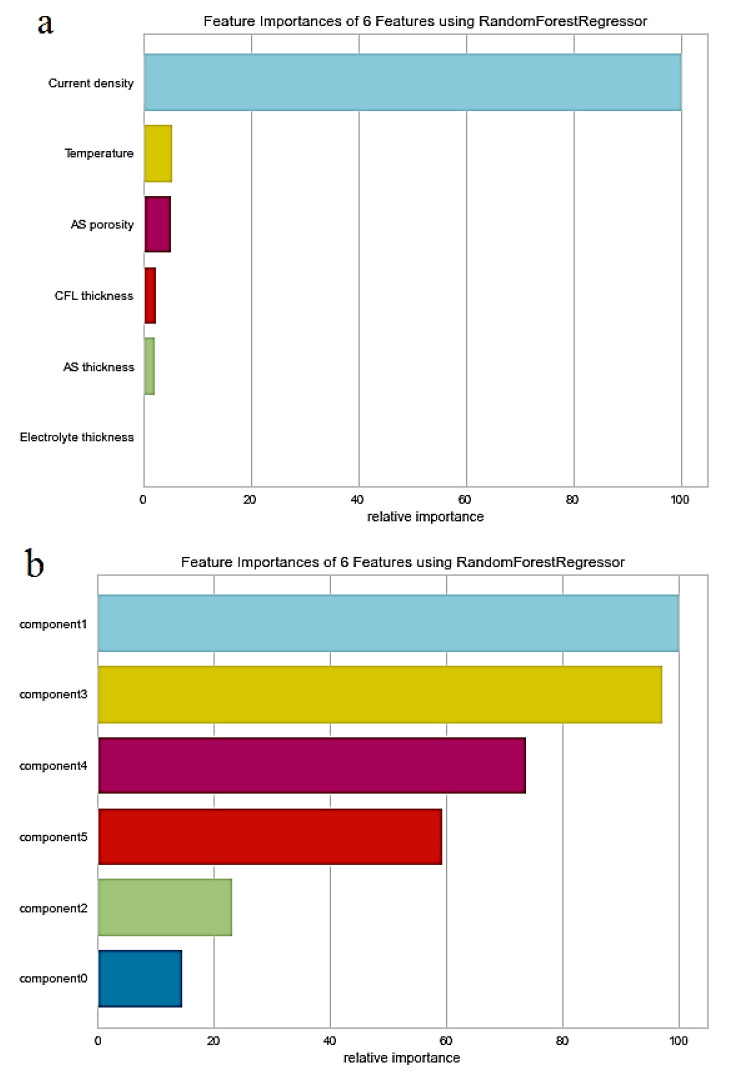
Random forest importance of (**a**) standard original features and (**b**) decomposed features using PCA.

To measure the features’ importance, the RandomForest feature_importances_ attribute from the scikit-learn library was used and visualized with feature importances from the yellow brick library. After using PCA, the importance of the features was changed because of mapping the features to some new components with nearly zero correlation. The features’ importance using random forest before and after using PCA is shown in Figure 4. It shows that the current density has the most effect on voltage changes. So, it can be accepted because the experiments also showed that current density influences the voltage more than other parameters.

### 2.7. Features and Target Correlation Inspection

**Figure 5:**
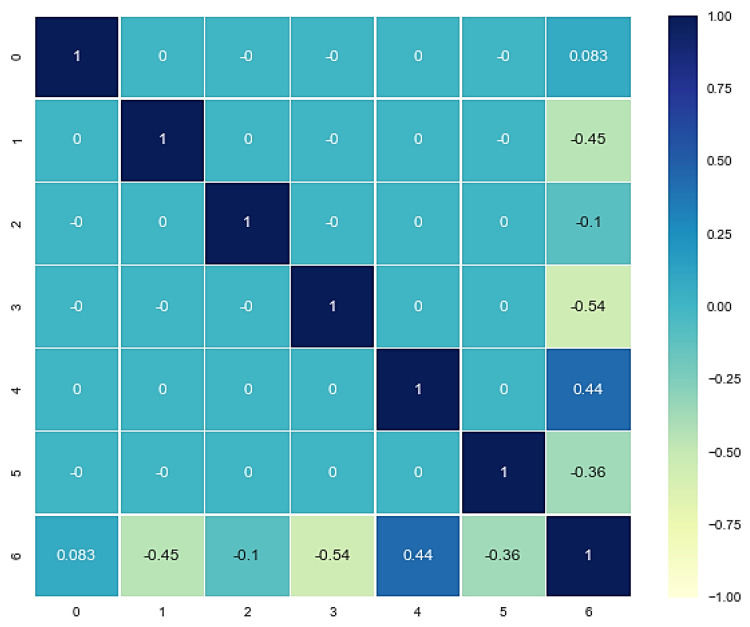
The correlation heatmap between the components and target.

The correlations between the features and target are essentially significant in choosing the right and proper model. The Pearson correlation between the features and target can be in the range of −1 to 1, while the correlation value close to 0 indicates the nonlinear relation between the features and the target. A correlation value close to −1 or 1 indicates that there is a linear relationship between the features and target (close to 1 indicates direct and −1 indicate inverse trend). It can also be interpreted that the low correlation absolute value between the features and target (|X,ycorr| ≈ 0) indicates high complexity, and therefore a complex model is desired. The heatmap of Pearson correlations between the features and target is shown in Figure 5.

### 2.8. Splitting Data into Train and Test Data

After preprocessing procedure, the data is split into train and test sets. The models are first fitted on train data and finally evaluated with test data which are unseen for models. For this purpose, the train_test_split function was used from the scikit-learn library to split data. To evaluate the model with unseen data, test_size was set as 0.2. It means 80% of the data was used as training data, and the remaining 20% was used as test data to evaluate the learning models. Splitting the data into train and test sets were done as shown below:
from sklearn.model_selection import train_test_splitX_train,X_test,y_train,y_test = train_test_split(X,y,test_size = 0.2,random_state = 10)

### 2.9. Building a Dummy Model

As a baseline to compare the model scores with, the Dummy regressor model was fitted to the training data. The Dummy regressor is a model with a straightforward strategy for target prediction without considering the relations between the features and target value. It predicts the unseen data with some simple strategy such as mean, median, or most frequented. Dummy regressor was fitted on the train data as shown below:
from sklearn.dummy import DummyRegressordr = DummyRegressor()dr.fit(X_train,y_train)

### 2.10. Plotting Learning and Validation Curves

To evaluate the effect of the number of samples on the learning rate of the model and the significance of the regularization parameters, the learning and validation curves were plotted by using the learning curve and validation curve from the yellow brick library. For example, for the mentioned KNN regression model, as shown below, the learning and validation curves were plotted using the following codes:
from yellowbrick.model_selection import ValidationCurve, LeaningCurvevc = ValidationCurve(model,param_name = “n_neighbors”,param_range = np.arange(1,20), cv = cv, scoring = “r2”)vc.fit(X,y)vc.show()

### 2.11. Plotting Prediction Error

Plotting prediction error plot can be helpful by plotting the actual targets against the predicted targets in one diagram. The model generalization can be evaluated by comparing the distribution of the actual and predicted data on this plot and considering the R^2^ score. By comparing the distribution of the actual and predicted targets against the 45-degree line, the model can be evaluated. Plotting the prediction error plot of the mentioned KNN regressor model was done using prediction error API from the yellow brick library and is shown below:
from yellowbrick.regressor import PredictionErrormodel = modelvisualizer = PredictionError (model)visualizer.fit (X_train, y_train)visualizer.score (X_test, y_test)visualizer.show()

### 2.12. Metrics

To evaluate the built model on the train and test data, some regression metrics were used. These scores compute the deviation between predicted values and actual observation [28]. R^2^ score were used to return the regression score by considering the coefficient of determination (Equation (1)) and yield a score between 0 and 1. A value of 1 corresponds to a perfect prediction and the value of 0 means the model just predicts based on the mean of the training set. Mean squared error (MSE) and mean absolute error (MAE) were also used to measure the errors of the prediction (Equations (2) and (3)).

(1) $$R^{2}=1-\frac{-\sum^{\;}\left(Y_{i}-{\hat{Y}}_{i}\right)}{\sum^{\;}{\left(Y_{i}-{\hat{Y}}_{i}\right)}^{2}}$$

(2) $$\mathrm{MSE}\;=\frac{1}{n}\sum_{i=1}^{n}{\left(Y_{i}-{\hat{Y}}_{i}\right)}^{2}$$

(3) $$\mathrm{MAE}\;=\frac{1}{n}\sum_{i=1}^{n}{\left(Y_{i}-{\hat{Y}}_{i}\right)}^{2}$$

## 3. Model Selection and Hyperparameters Tuning

Two major groups of machine learning algorithms are supervised and unsupervised learning in material science. The supervised learning is divided into two subgroups: regression and classification [29]. The classification methods aim to predict a target’s class label [30]. It can be a binary or a multiclass system. The regression tasks aim to predict the continuous relations between inputs and outputs [31,32]. Linear regression (also called ordinary least squares), and kernelized models including support vector regressor (SVR) and Gaussian process regressor (GPR), K-nearest neighbors (KNN), decision tree regressor and its ensemble includes Random Forest regressor (RF) and gradient boosting regressor (GR); the K-nearest neighbors (KNN) and neural network algorithms were applied to the data to build the best model. 

### 3.1. Machine Learning Models

#### 3.1.1. Linear Regression

Linear regression or ordinary least squares (OLS) is a linear model and is one of the simplest models used in machine learning prediction models. It makes predictions using a linear function of the features. The general prediction formula of the linear models is given in Equation (4).

(4) $$\;\hat{Y}\;\cdot =\cdot w\left[0]\cdot \times \cdot x[0]\cdot +\cdot w[1]\cdot \times \cdot x[1\right]\cdot +\ldots \cdot +\cdot w\left[p\right]\cdot \times \cdot x\left[p\right]\cdot +\cdot b$$

where *p* is the number of the features for one sample, ‘w’ and ‘b’ are the learning parameters of the model, and ŷ is the model prediction. The linear regression tries to find the ‘w’ and ‘b’ and minimize the ‘mean squared error’ between the predicted and actual regression target. The ‘mean squared error’ is the sum of the predicted and actual regression target differences. Linear regression has no tunable hyperparameter, making it easy to use; however, there is no control over the model complexity.

#### 3.1.2. Kernelized Models

##### Support Vector Machine (SVM)

Linear models often have limited flexibility, and as a result, it is hard to separate the data in a non-linear model. Some tricks are often used to improve the flexibility of the linear models, called kernel tricks. Two kernel tricks are commonly used in support vector machines named Polynomial kernel and radius basis function (RBF) kernel, also known as the gaussian kernel. Using these functions are helpful to map the data in higher-dimensional space and make the decision easier for the model. Support vector machines can solve linear problems with non-linear solutions. During the training, the SVM learns the importance of each data point to represent the decision boundary between the samples. These decision boundaries determine the sample’s border, called support vectors. In the prediction step, the distance of each new data point to each support vector is measured, and the new datapoint is predicted based on mentioned support vectors [33]. The distance between data points is measured by the Gaussian kernel, as shown in Equation (5):

(5) $$k_{\mathrm{rbf}}(x_{1},\;x_{2})=\exp\;(\gamma ||x_{1}\;-\;x_{2}|{|}^{2})$$

the *x*_1_ and *x*_2_ are the data points, ||*x*_1_ − *x*_2_||^2^ is the Euclidean distance, and the gamma is the parameter for regularizing the Gaussian kernel width.

Support vector machines have some advantages and disadvantages. SVMs usually work well on low and high-dimensional data (dimensions can be considered the number of the features). It can also perform well on data with high dimensions and few samples. On the other hand, SVMs are very sensitive to careful preprocessing of the data and tuning of the hyperparameters. However, the linear or tree-based models, such as random forest or gradient boosting, may not require preprocessing or need little preprocessing [34].

##### Gaussian Process

The Gaussian process model is a non-linear model based on Gaussian process priority to make regression predictions. For the best prediction, all the variables should obey the Gaussian distribution [35]. This model has advantages such as different kernels and high flexibility on different data to capture a large complexity range. The main difference between the Gaussian process and the other kernelized model (SVM) is that this model has an iterative algorithm, works with linear and non-linear kernels, and solves the problems using different kernels iteratively. However, in the case of the SVM, the model has a linear algorithm and solves the linear problems using kernel tricks such as polynomial [36]. Different kernels and related functions are used in the Gaussian process model represented in Table 1. The performance of the Gaussian process model is determined by the mean and covariance functions.

#### 3.1.3. Decision Tree and Ensembles

**Figure 6:**
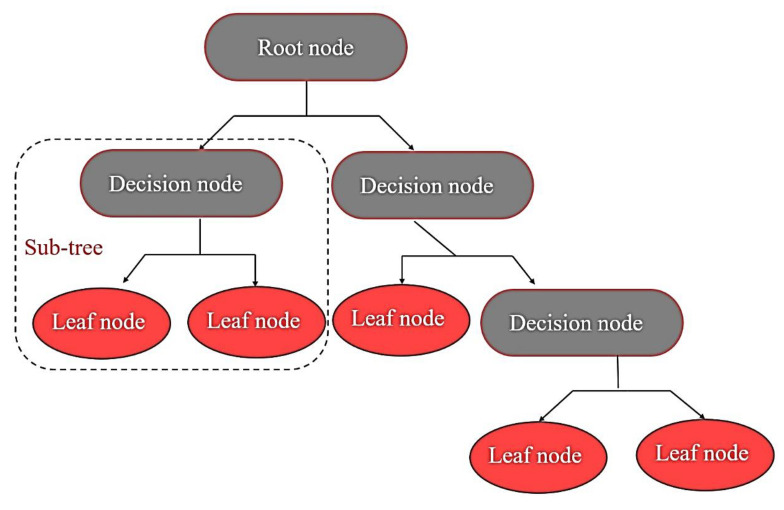
The schematic of decision-making in decision tree.

Decision trees are widely used models for classification or regression problems. In these models, the learning is performed through hierarchy if/else questions to lead a decision-making. These if/else questions are similar to the asked questions in the 20 Question game to distinguish the correct answer. The decision tree structure is quite simple and shown in Figure 6. The top node, called the root, represents the whole dataset, internal nodes represent the dataset’s features, branches represent the decision rules, and each leaf node describes the outcome. In machine learning, these questions are called tests. The recursive questions process yields a tree of decisions and is repeated until each leaf in the decision tree only contains a single target value (single regression value).

Prediction of new data is made by checking the new data point lies on which partition of the feature space and then predicting the majority target in that region. The region can be found by traversing the tree based on the test in each node and finding the exact leaf in which the new data point falls. The prediction can be made by measuring the mean targets of the related leaf.

Compared to other algorithms, decision trees have some advantages over many algorithms: the results can be easily visualized and understood by non-experts, and the algorithms are invariant to the data scaling. It can be used without any normalization or standardization. It is also suitable for mixed binary and continuous features. On the other hand, the main downside of decision trees is that they have a tendency for instability and provide poor generalization performance. The ensemble methods based on decision trees discussed next section could be used to overcome this problem.

Ensemble methods based on decision trees are powerful models which combine multiple decision tree models. The downside of the decision tree model is its great potential for instability. In most cases, it has high variance and bias, which is not favored for machine learning models. Ensemble methods based on the decision tree were used to overcome this defect and reduce the variance or bias of the model. Two ensemble methods that have proven to be effective are random forest and gradient boosting methods; both use decision trees as their blocks.

Random forest is a collection of decision trees, where each tree is independent and makes decisions independently. The overfitting reduction concept of random forest is related to the fact that each tree might work well and likely overfit on the part of the data. Overfitting could be reduced by averaging the result of all the trees. Bootstrap sampling is used to make a sample for the random forest. It means that a subset of the n_samples of the dataset can be randomly drawn and fed to the random forest. Each tree fits on a random subset, and the final prediction is the average of all the trees. The random forest overfits less than any of the trees in the model. The more trees in the model, the smoother the boundaries achieved in the prediction [37].

Another ensemble method based on decision tree is gradient boosting. Multiple decision trees are combined in gradient boosting to build a gradient boosted model. In this case, the model works by serially building several trees, and each tree tries to modify the previous tree’s mistake. In contrast to the random forest, there is no bootstrapping in gradient, but strong pre-pruning is needed.

#### 3.1.4. Nearest Neighbors

The k nearest neighbor (KNN) is one of the oldest algorithms among all machine learning methods. It has a simple strategy to make a prediction, which considers the nearest neighbors around the new data point and makes the prediction. There are two crucial adjustable parameters to optimize the algorithm: the number of neighbors and the way of distance measurement. KNN has some advantages and disadvantages. It is straightforward to understand and usually works appropriately without any adjustments. Nevertheless, the downside of this model is the slow prediction of the model. In some applications, time is an important parameter and KNN may not work efficiently on large datasets [34].

#### 3.1.5. Neural Network

The first neural network was introduced based on the biological neuron system in animal brains to perform complex computations. Then, various architectures were designed, as we will see. The neural network is based on connected neurons which build the model. All the neurons are connected, and the information is transferred through these connections. A well-known neural network in machine learning is the multilayer perceptron (MLP) model. Some other neural network models include convolutional neural network (CNN), which is often used for image processing, and recurrent neural network (RNN), which is usually used for natural language processing. This study uses the MLP models to evaluate the neural network models on the dataset [38].

The significant advantage of the neural network is its ability to capture information in a large amount of data and build a very complex model. With enough time, computational resources, and careful parameter tuning, the neural network beats other machine learning algorithms. The main downside of the neural network is that it is tough to tune the parameters. It is also a black-box model, and we have limited control over the model [39].

### 3.2. Hyperparameters Tuning

#### 3.2.1. Linear Regression

As mentioned in the previous section, Linear regression is straightforward to work because it has no hyperparameter, and therefore there is no control on the model complexity by hyperparameter tuning. So, we just fit the linear regression to the train data with the following codes:
from sklearn.linear_model import LinearRegressionlr = LinearRegression()lr.fit(X_train,y_train)

#### 3.2.2. Support Vector Machine

In contrast to linear models, SVMs have tunable parameters which used to optimize the model. ‘kernel’, ‘C’, and ‘gamma’ are the essential parameters tuned. Four kernels named linear, poly, RBF, and sigmoid were used in support vectors to solve the problems in different spaces. ‘C’ is the regularization parameter that controls the importance of each data point during support vectors. Increasing the ‘C’ parameter value increases the model complexity, and the model’s complexity can be adjusted by choosing the proper ‘C’ value. Control over the Gaussian kernel width can be performed by tuning the parameter ‘gamma’.

All the parameters were tuned using gridsearchCV from the scikit-learn library. The support vector regressor model was fitted on train data, and the best hyperparameters were evaluated using the following codes (the parameter ‘gamma’ was used as default):
from sklearn.svm import SVRsvr = SVR()cv = ShuffleSplit (n_splits = n,random_state = 10)gscv = GridSearchCV(svr, cv = cv, n_jobs = −1, param_grid = {‘kernel’:[‘rbf’,’sigmoid’,’poly’,’linear’], ‘C’: np.arange(n,m,0.1)}, verbose = 5)gscv.fit(X_train,y_train)

#### 3.2.3. Gaussian Process

Some essential hyperparameters should be tuned to fit the Gaussian process model on train data. The gaussian hyperparameter tuning is a hard problem due to its difficulty and sensitivity to initial values of the parameters such as alpha and optimizer. The complexity of the model could be tuned by modifying the alpha parameter. The four mentioned kernels have specified parameters represented in Table 2.

The model fitted on-train data with Matern kernel and tuned the hyperparameter as shown below:
from sklearn.gaussian_process import GaussianProcessRegressorfrom sklearn.gaussian_process.kernels import DotProduct, Maternkernel = Matern(length_scale = n,nu = m)gpr = GaussianProcessRegressor(kernel = kernel, random_state = 0, alpha = k)gpr.fit(X_train,y_train)

##### Decision Trees

Building a tree on data and continuing until all leaves have become pure (containing only one target value) leads to a model with highly overfitting training data. There is a strategy in scikit-learn to prevent overfitting: stopping the creation of a tree early (pre-pruning) by limiting the maximum depth of the tree, limiting the maximum number of leaves, or requiring a minimum number of points in a nod to keep splitting it. The following codes were used to fit the model on train data before any pre-pruning:
from sklearn.tree import DecisionTreeRegressordr = DecisionTreeRegressor(random_state = 0)gscv = GridSearchCV(dr, cv = cv, param_grid = {‘max_depth’: np.arange(n,m,1)}, verbose = 5)gscv.fit(X_train,y_train)

##### Random Forest

Two primary hyperparameters of the random forest model were evaluated: ‘n_estimator’ and ‘max_features’. The number of built trees is controlled by adjusting the value of ‘n_estimator’. The ‘max_features’ determine the randomness of the trees. The smaller value for ‘max_features’ usually reduces overfitting. Usually, for regression problems, ‘max_features’ is set as log2(n_features) to lead to the best performance. Increasing the value of ‘n_estimator’ also leads to a more powerful ensemble model because averaging on more trees can reduce the overfitting of the model more efficiently. The random forest regressor was fitted on train data as shown below:
from sklearn.ensemble import RandomForestRegressorrr = RandomForestRegressor(n_estimators = n)rr.fit(X_train,y_train)

##### Gradient Boosting

Gradient boosting, as described previously, is an ensemble method of decision trees. Some parameters can be adjusted, but in this work, the n_estimator is just adjusted to optimize the model generalization. The model, like the other mentioned models, fitted on train data as shown below:
from sklearn.ensemble import GradientBoostingRegressorgbr = GradientBoostingRegressor(n_estimators = n)gbr.fit(X_train,y_train)

#### 3.2.4. Nearest Neighbors

The number of neighbors is the main hyperparameter that can be adjusted in the KNN regressor. Increasing the number of neighbors leads to a decrease in the model complexity. On the other hand, the minimum value (1) increases the model complexity. There are other parameters such as ‘weights’, but the number of neighbors has a major role. The model fitted on train data using the following codes:
from sklearn.neighbors import KNeighborsRegressorgscv = GridSearchCV(knn,param_grid = {‘n_neighbors’: range(n,m)}, cv = cv, n_jobs = −1, verbose = 5)

#### 3.2.5. Neural Network

The neural network used in this work was multilayer perceptron (MLP) from scikit-learn library. Some hyperparameters such as hidden layer size, the number of neurons, alpha, activation, and the solver can be adjusted to optimize the model generalization. Increasing hidden layer size and the number of neurons in each layer increases the complexity of the model. Alpha is the dropout parameter that disconnects the connections of neurons from one layer to the next. Activation and solver can be adjusted with respect to the problems and dataset. In this study, the MLPs neural network is fitted to train data with specific hyperparameters, as shown below:
from sklearn.neural_network import MLPRegressornnr = MLPRegressor(hidden_layer_sizes = (n,m), activation = ‘activation function’, random_state = 0, solver = ’solver’, alpha = k)nnr.fit(X_train,y_train)

**Figure 7:**
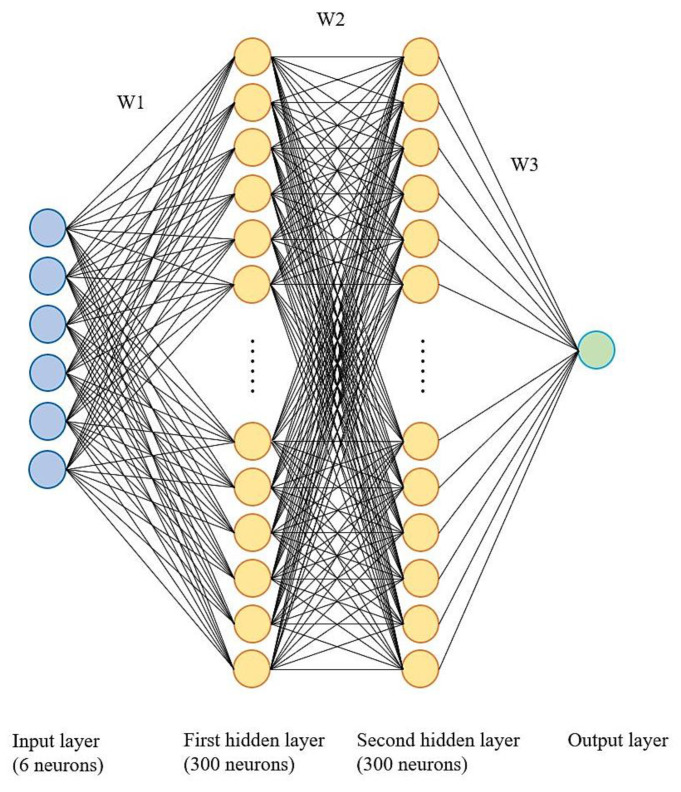
The structure of multilayer perceptron neural network used. W1, W2, and W3 are three weights matrixes that determine each neuron’s output.

The structure of the neural network model used illustrate in Figure 7.

More information about the dataset and implemented sources are available in supplementary (Appendixes SA and SB).

## 4. Results and Discussion

In order to measure the prediction score of the regression models, the R^2^ score (0 < R^2^ < 1) was used. The higher the value of R^2^ indicates a more accurate prediction for the model, and the lower value means a reduced ability of the model. All the models were trained with a training set as mentioned in the previous section and the R^2^ score was measured. To evaluate the models’ errors, some error metrics such as mean absolute error and mean squared error were used. A comprehensive evaluation of all models was performed, and the result is represented in Table 3.

The value of R^2^ and errors represented in Table 3 for different models showed that the best machine learning model for predicting the relations between the dataset variables is a multilayer perceptron (MLP). Multilayer perceptron model has the highest R^2^ and the lowest mean squared and absolute error. This model with perfectly tuned hyperparameters had good generalization. On the other hand, the lowest R^2^ and the highest mean squared and absolute errors are related to the linear regression model. These scores could be expected because, as mentioned before, the correlation value close to 1 or −1 could be interpreted as a linear relationship between two variables. In contrast, the correlation value close to 0 could be construed as non-linear relation between two variables. By considering the relations between the features and the target (Figure 5), it can be interpreted that the relations between the features and the target are mostly non-linear since the essential components (after using PCA, the features mapped onto new components which carry same information for model) have a correlation value of −0.54, −0.45, 0.44, and −0.36 with the target, respectively. Therefore, it was observed that all the features have non- and semi-linear relation with the target value. It was found that linear models consider a linear function to predict the targets. So, it seemed that the linear model could not learn the relations properly. In the neural network case, with perfect hyperparameters tuning, the model could learn the relations excellently. However, it usually works well on high-volume data. The other models, i.e., kernelized models (support vector regressor (SVR) and Gaussian process(GP)), decision tree and its ensembles (RF and GB), and K-nearest neighbors predict the hidden function of the data reasonably. Nevertheless, the decision tree is an unstable model with high bias or variance having the highest error among the other models. This model seemed to be underfitted or overfitted. Ensembles of the decision trees may reduce the errors and improve the R^2^ score.

**Figure 8:**
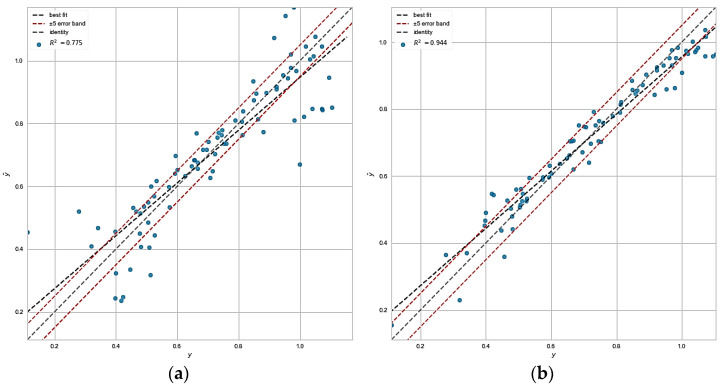

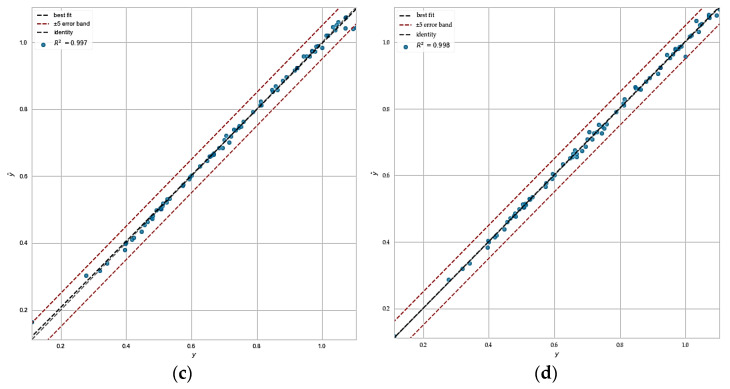
Different machine learning models’ prediction error on test data for (**a**) linear regressor; (**b**) support vector regressor; (**c**) Gaussian process; and (**d**) multilayer perceptron.

Prediction error plots were used to measure the ability of the models in prediction and generalization, as shown in Figure 8. The higher prediction accuracy is related to the graph in which the predicted values are closer to 45° line. It other words, the dispersion between the predicted and test target data is small, and these two values are close. The highest R^2^ score is related to the MLP neural network, which exhibits the best generalization. The highest dispersion is related to the linear regression model, and then to the decision tree (Figure S1e–h). Other models showed low dispersion in prediction error plots. The Gaussian process regressor is the best model after MLP neural network due to its flexibility in different complexity and the strong potential to find complex relations between the data. The decision tree model exhibits the highest dispersion, which means the lowest R^2^ score after linear regression. Low generalization of the model can be seen in the prediction error visually. Random forest and gradient boosted trees exhibit better dispersion close to 45° line compared to the decision tree. The key factor in the experimental dataset is the bias during the experiments, such as human error, experiment error, or calculation error. Therefore, the bias of the model can be reduced by using the gradient boosting model. This correction is due to modifying a series of decision trees in which each tree tries to modify the previous one. So, in this dataset, the probability of bias is high since the data was collected from an experiment. Hence, a gradient boosting model could be more efficient than a random forest model, which was also proved by the corresponded R^2^. In the case of K-Nearest neighbors, this is a classic and non-parametric model, and it could be either linear or non-linear based on data, and this R^2^ score is not unexpected.

**Figure 9:**
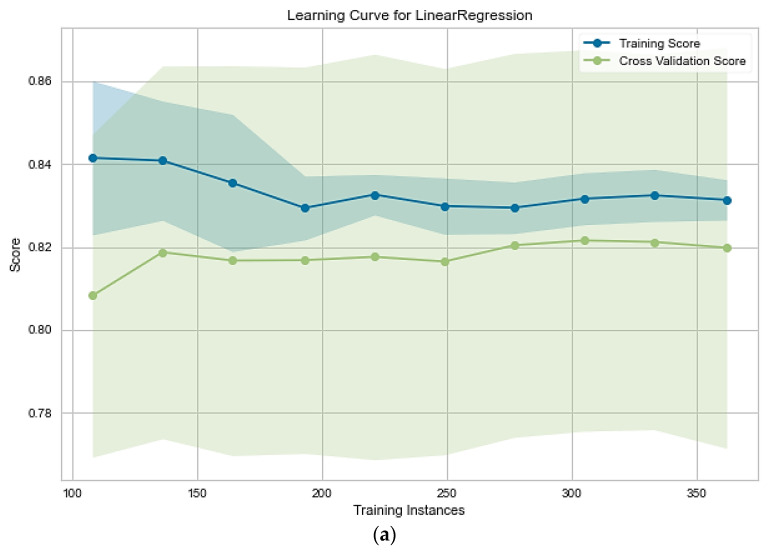

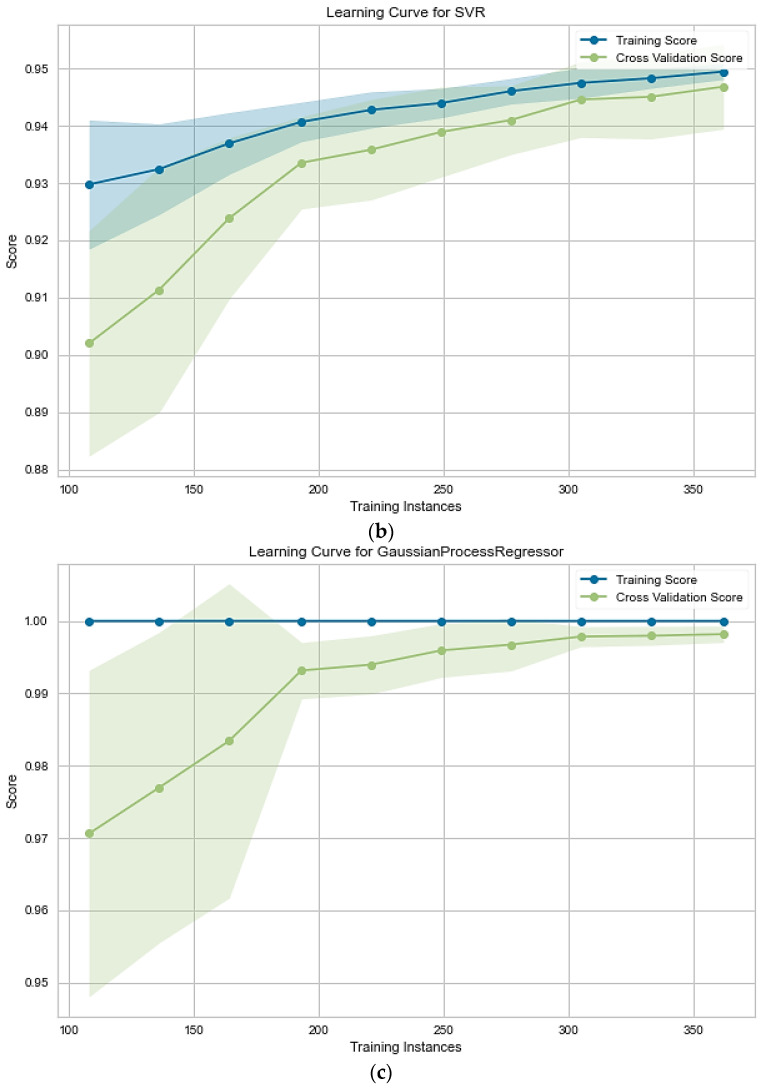

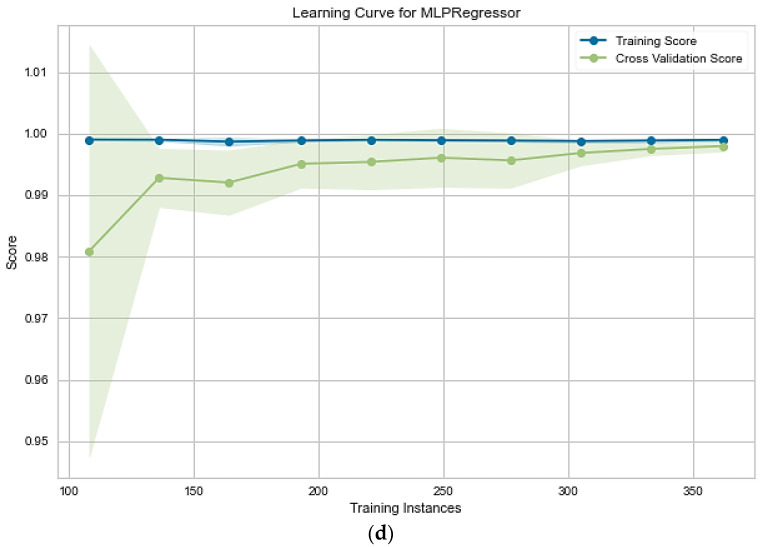
Different machine learning models’ learning curve for (**a**) linear regression; (**b**) support vector regressor; (**c**) Gaussian process; and (**d**) multilayer.

It is worthy to note that the number of samples can significantly affect models’ accuracy and generalization. The more samples, the better the accuracy score and generalization are expected. In some cases, however, a higher number of samples is helpful for a model, though the model then may require more powerful resources such as Central processing unit (CPU), memory, and graphics processing unit (GPU). Hence, learning curve plots were used to measure the effect of the number of samples on the trained machine learning models, and the results are shown in Figure 9. The lines are the mean score value, and the shaded area around each lines indicate the variance of the model.

According to Figure 9, the linear model score was not influenced by the number of samples. It is reasonable because, with an increase in the number of samples, the relationship between variables defiantly did not tend to be linear. Therefore, it could be concluded that the complexity of the problem is higher than the linear model complexity. In all other cases, increasing the number of samples results in an improvement in models’ scores (Figure S2e–h). In other words, increasing the number of samples could provide ample information space for the model because each new sample contained new information. Therefore, for the appropriate model, further data supplementation in this system could be helpful until the cross-validation line reaches a stable state. After the semi-horizontal trend in the cross-validation line, increasing the number of samples is not reasonable because the extensive data needs more robust resources and increased calculation expenses. It should be mentioned that the linear algorithms in the linear regressor and SVR led to a lower cross-validation score than other non-linear models because of the non-linear relations between features and target value, which were discussed earlier. Hence, it can be concluded that the number of samples was enough to build the general models. These results, therefore, approved the reliability of built general models in this study.

During the increase in the sample amount, the variance of the models’ prediction became smaller. It is due to the information that the samples carried and gave the generalization possibility to these models. In the first step of the learning curve, the two best models, the Gaussian process, and MLP neural network start with high variance. During the training phase, the variance gradually decreased; in the final step, the variance reached the smallest value. The neural network model could predict the targets with about 250 training samples in this case. This model captured all the relations in just about 250 training samples. The model accuracy did not improve during the increase in the training samples.

**Figure 10:**
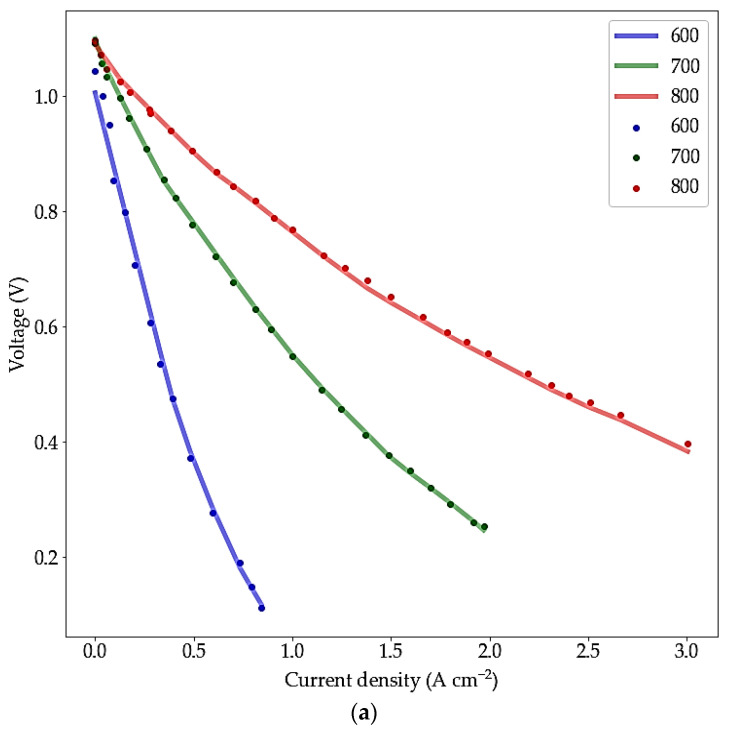

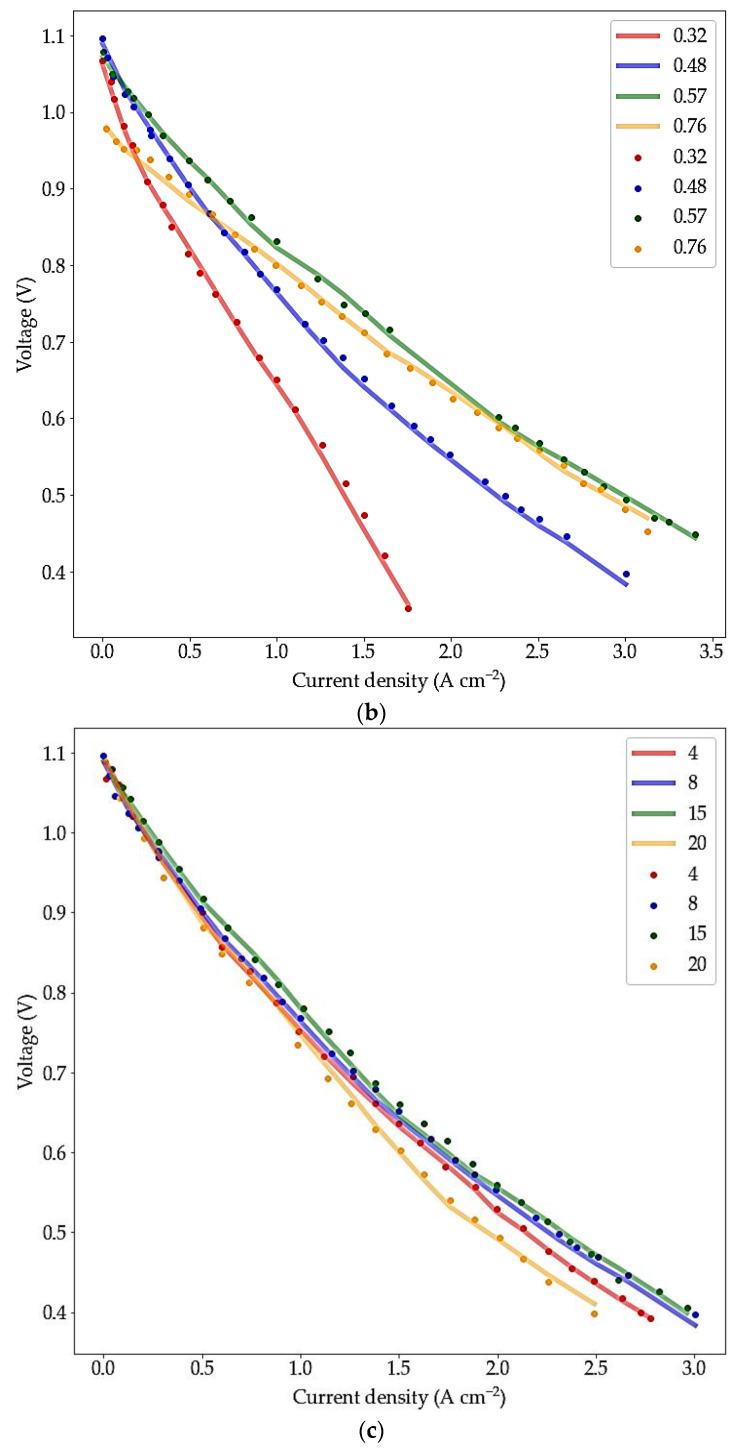

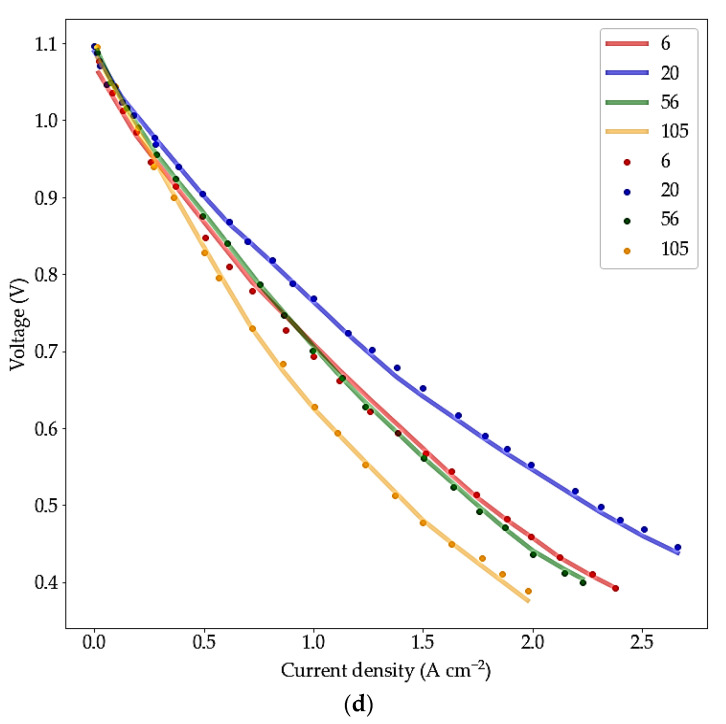
MLPs prediction and experimental data of cell current–voltage dependency at (**a**) different operation temperatures; (**b**) different ASL porosity (at 800 °C); (**c**) different electrolyte thickness (at 800 °C); and (**d**) different CFL thickness (at 800 °C); the scattered dots related to the experiments data points, and the lines related to the model prediction trends.

The main advantage of ML over utilizing mathematical modeling is its convenience and accuracy. A mathematical model proposed by AI. Milewski [23] predicted the behavior of an anode-supported cell from experimental data presented by Zhao and Virkar in 2005 [24]. Equation (6) was introduced by this model to represent the voltage–current dependency in a variety of physical and operational conditions.

(6) $$E_{SOFC}=\frac{E_{max}-\eta_{f}.i_{max}.r_{1}}{\frac{r_{1}}{r_{2}}\left(1-\eta_{f}\right)+1}$$

where $E_{max}$ is maximum voltage, $\eta_{f}$, $i_{max}$, $r_{1}$, and $r_{2}$ are fuel utilization factor, maximum current density, area specific internal ionic resistance, and area specific internal electronic resistance, respectively. Combining laws and relations, such as the electrical laws, solid material properties, and some electrochemical laws, must be considered to drive this kind of model and the combination requires assumptions and a wealth of knowledge. Therefore, these mathematical models are not flawless in prediction, and it is challenging to propose a mathematical model regardless of assumptions. For example, the model proposed by Ref. [23] assumed the independency of temperature on the electrical resistivity, which causes a difference between the mathematical model predictions and experimental results. However, utilizing the ML method with different algorithms can increase accuracy and reduce calculation costs. The MLP model proposed in this paper predicts the effect of temperature, anode porosity, electrolyte, and CFL thickness on the current–voltage dependency with 1%, less than 1%, 1%, and less than 1% percentage error values, respectively. The resulting plots are shown in Figure 10a–d. The changes imposed by variation in the abovementioned parameters showed reasonable compatibility with the literature. Briefly, the elevation of temperature results in increasing the output voltage at a constant current due to an increase in Ni/YSZ conductivity [40]. Additionally, the anode porosity plays an effective role on output voltage in the same situation. By increasing the porosity to an optimized value, the output voltage increases because of the gas diffusion facilitation and proper distribution of the active surface area [41,42].

According to Figure 10, the predicted results followed the experimental data properly. The relative error values proposed by ref [33] for the temperature, anode porosity, electrolyte, and anode thickness were in the range of 2 to 7%. It is worth mentioning that the ANN prediction for this dataset in ref [12] illustrated an imprecise prediction line for 32% porosity within the anode while according to Figure 10, the MLP model in this paper showed a more accurate prediction.

## 5. Conclusions

Machine learning method has shown numerous benefits, such as saving time and energy within the long-term experimental procedures and being cost-effective, making it an appealing approach for prediction in various study fields. Therefore, this method is a viable approach to evaluating solid oxide fuel cells due to their complex fabrication process and operational conditions. Regarding this paper, eight machine learning models were conducted to compare their accuracy for predicting the output voltage of an anode-supported solid oxide fuel cell, including linear regressor, K- nearest neighbors regressor, support vector regressor, random forest regressor, gradient boosting regressor, Gaussian process regressor, and multilayer perceptron regressor. The latter was implemented with two hidden layers, Relu activation function, and 300 neurons for each hidden layer. The results were discussed according to three metrics, mean absolute error, mean squared error, and R^2^ score, which evaluated both the models’ error range and accuracy. It was indicated by the results that the complex models such as multilayer perceptron and Gaussian process regressor provided higher accuracy due to the non-linear correlation between the features and target values; on the other hand, linear and support vector regressors could not perform an efficient prediction because of their linear solver and low complexity. The multilayer perceptron regressor showed the highest R^2^ score among the aforementioned models with a 0.998 R^2^ score, and the lowest mean absolute and squared error of 0.006 (V) and 9.6 × 10^−5^, respectively. The Gaussian process regressor yields an R^2^ score of 0.996, mean absolute, and squared error of 6 × 10^−3^ (V) and 10^−4^, respectively. Therefore, regarding these observations, the MLP regressor is a robust model able to predict the output voltage of an anode-supported solid oxide fuel cell based on its operational temperature, anode-supported porosity and thickness, electrolyte and cathode functional layer thickness, and current density. Afterward, the MLP method was conducted to predict the interrelations between features and current–voltage dependency, and the results indicated that the model prediction lines followed the experimental data prosperously and these prediction lines are more accurate than previous studies.

## Figures and Tables

**Table 1:** Gaussian process kernels with related functions.

Kernels	Function
DotProduct	$k(x_{i},x_{j})=\sigma_{0}^{2}$+ x_i_.x_j_
Matern	$k(x_{i},x_{j})=\frac{1}{\Gamma \left(\upsilon \right)2^{\upsilon -1}}$($\frac{\sqrt{2\upsilon }}{l}\;d\left(\mathrm{xi},\mathrm{xj}\right)$)^υ^K_υ_($\frac{\sqrt{2\upsilon }}{l}\;d\left(\mathrm{xi},\mathrm{xj}\right)$)
RBF	$k(x_{i},x_{j})=\exp\;(-\frac{d{\left(\mathrm{xi},\mathrm{xj}\right)}^{2}}{2l^{2}}$)
RationalQuadratic	$k(x_{i},x_{j})=(1+\frac{d{\left(\mathrm{xi},\mathrm{xj}\right)}^{2}}{2\alpha l^{2}}$)^-α^

**Table 2:** The gaussian process kernels’ hyperparameters.

Kernels	Parameters
DotProduct	Sigma_0
Matern	Length_scale, nu
RBF	Length_scale
RationalQuadratic	Length_scale, alpha

**Table 3:** Prediction accuracy and error of machine learning models.

Machine Learning Model	Train Score	Test Score (R^2^)	Mean Squared Error	Mean Absolute Error (V)
Linear model	0.843	0.774	0.012	0.078
Support vector (SVR)	0.949	0.944	0.003	0.041
Gaussian process (GP)	0.999	0.996	0.000	0.006
Decision tree	0.998	0.944	0.003	0.046
Random forest (RF)	0.997	0.984	0.000	0.020
Gradient boosting (GB)	0.999	0.984	0.000	0.021
K-nearest neighbors	1.000	0.985	0.000	0.016
Neural network (MLP)	0.998	0.998	0.000	0.006

## Data Availability

Not applicable.

## Supplementary Materials

The following supporting information can be downloaded at: https://www.mdpi.com/article/10.3390/ma15217760/s1, Figure S1a–d: Prediction error plots; Figure S2a–d: Learning curves.
