# Supplementary material for: Performance Analysis of Anode-Supported Solid Oxide Fuel Cells: A Machine Learning Approach
Source: Materials (Basel). 2022 Nov 3;15(21):7760. doi: 10.3390/ma15217760 (PMC9655730; doi:10.3390/ma15217760)
Supplement: Supplementary file 1 [file materials-15-07760-s001.zip › SupplementaryMaterial.pdf]

Supplementary material for

Performance analysis of anode-supported solid oxide fuel cells: A machine learning approach

Mohammad Hossein Golbabaei 1,*, Mohammadreza Saeidi Varnoosfaderani 2, Arsalan Zare 1, Hirad Salari 1, Farshid Hemmati 1, Hamid Abdoli 3 and Bejan Hamawandi 4,*

1 School of Metallurgy and Materials, College of Engineering, University of Tehran, Tehran 1417935840, Iran

2 School of Metallurgy and Materials Engineering, Iran University of Science and Technology,

Tehran 1684613114, Iran

3 Renewable Energy Research Department, Niroo Research Institute (NRI), Tehran 1468613113, Iran

4 Department of Applied Physics, KTH Royal Institute of Technology, SE-106 91 Stockholm, Sweden

* Correspondence: mh.golbabaei@ut.ac.ir (M.H.G.); bejan@kth.se (B.H.)

Prediction error plots

a

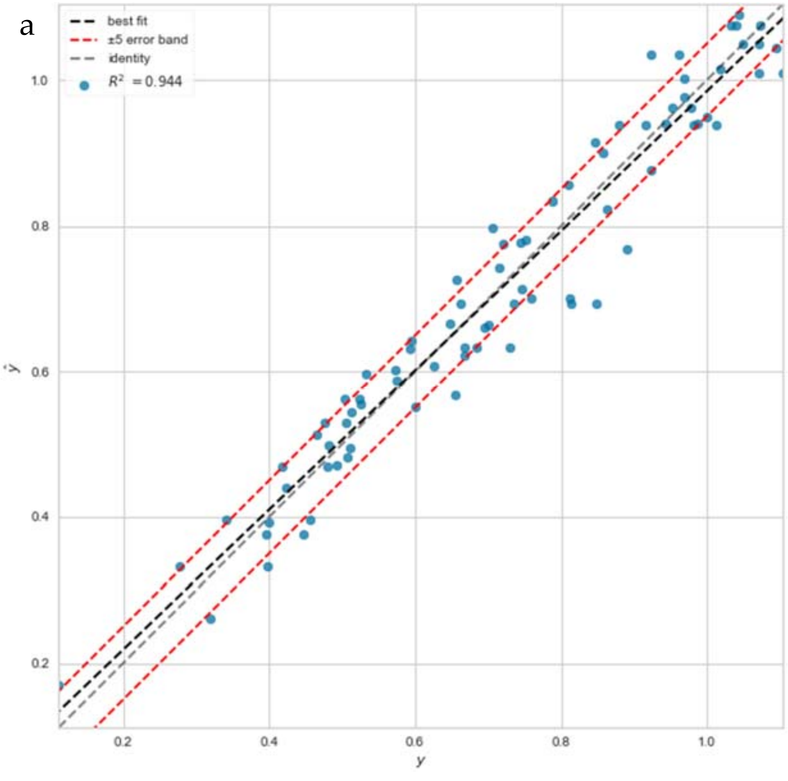

b

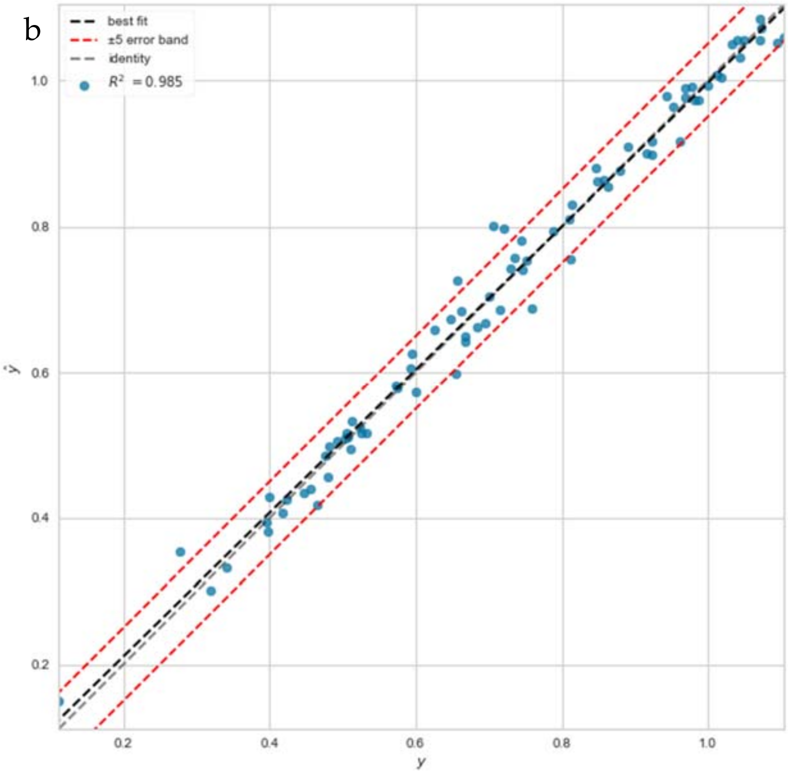
best fit
±5 error band
identity
R^2 = 0.985
0.2
0.4
0.6
0.8
1.0
ŷ
y

c

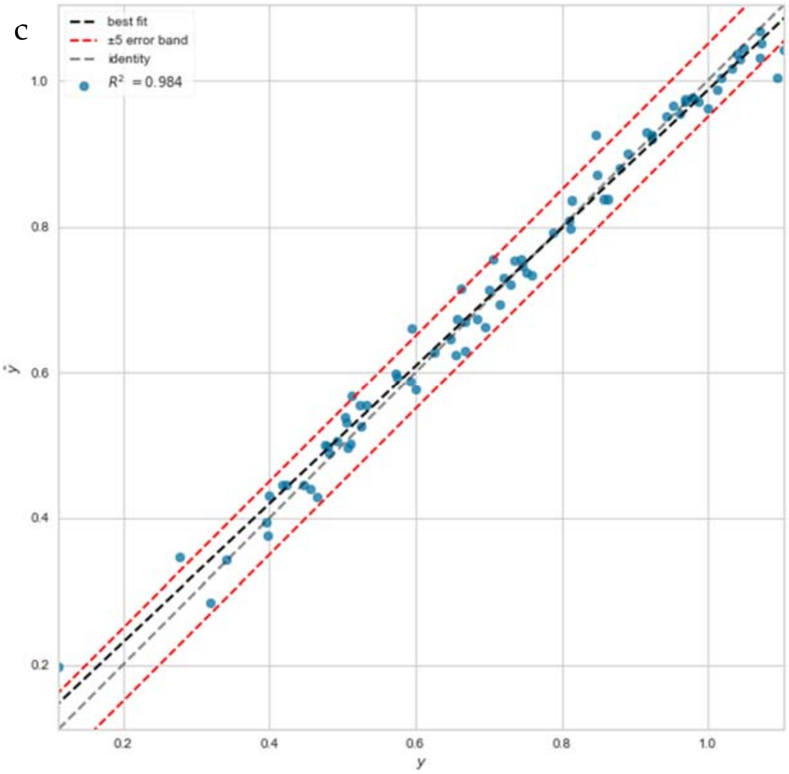
best fit
±5 error band
identity
R^2 = 0.984
0.2
0.4
0.6
0.8
1.0
ŷ
y

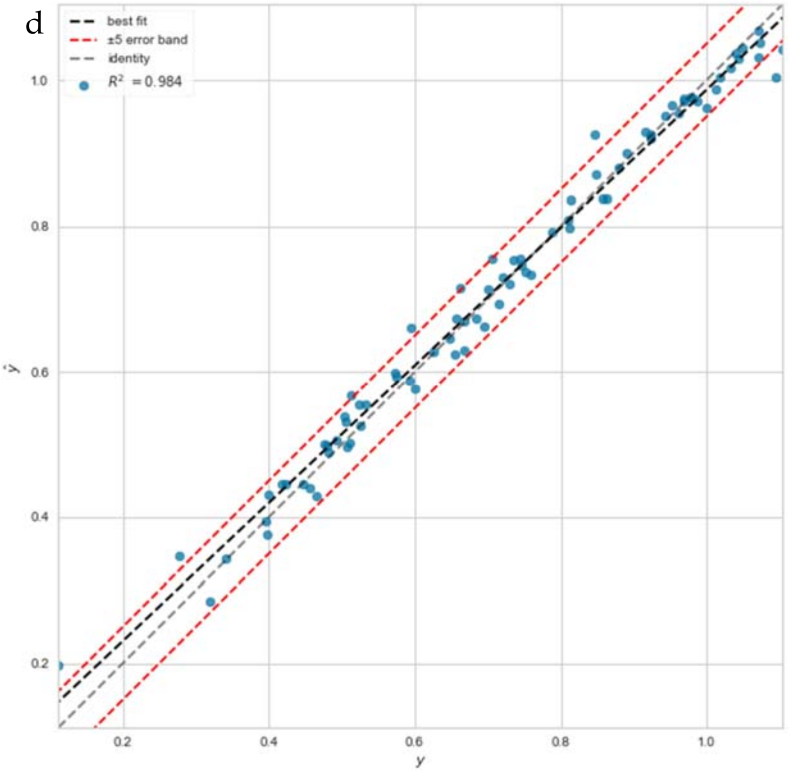

Figure S1. Different machine learning models' prediction error on test data for a) Decision tree regressor, b) KNN regressor, c) Random forest regressor, and d) Gradient boosting regressor.

Learning curves

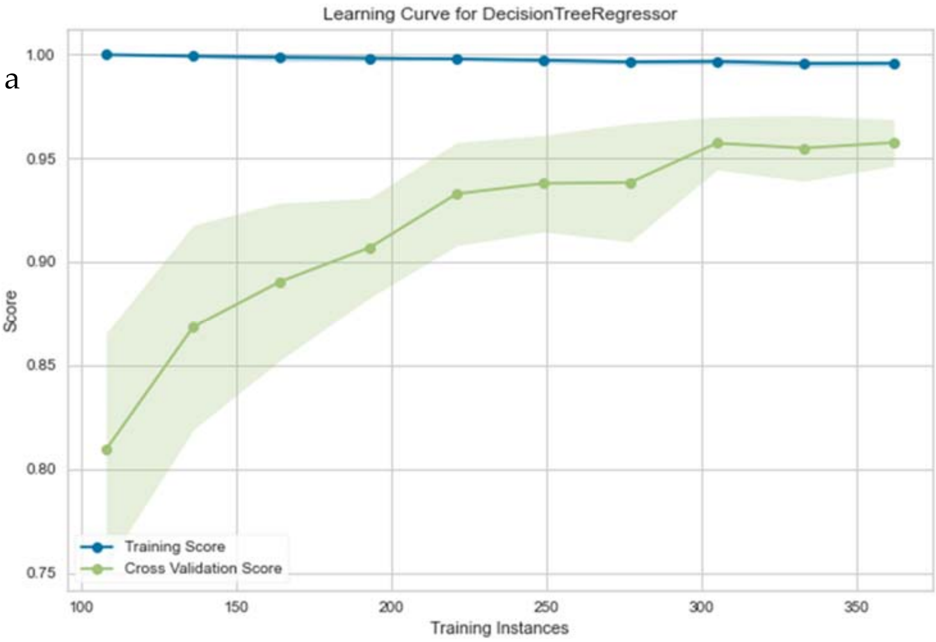

b

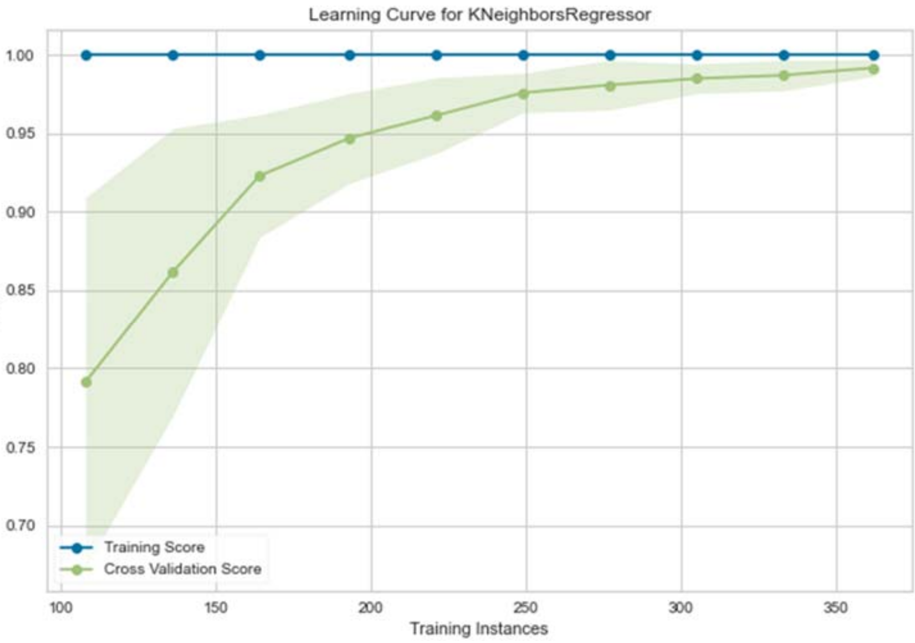

Learning Curve for KNeighborsRegressor
1.00
0.95
0.90
0.85
0.80
0.75
0.70
Score
Training Score
Cross Validation Score
100
150
200
250
300
350
Training Instances

c

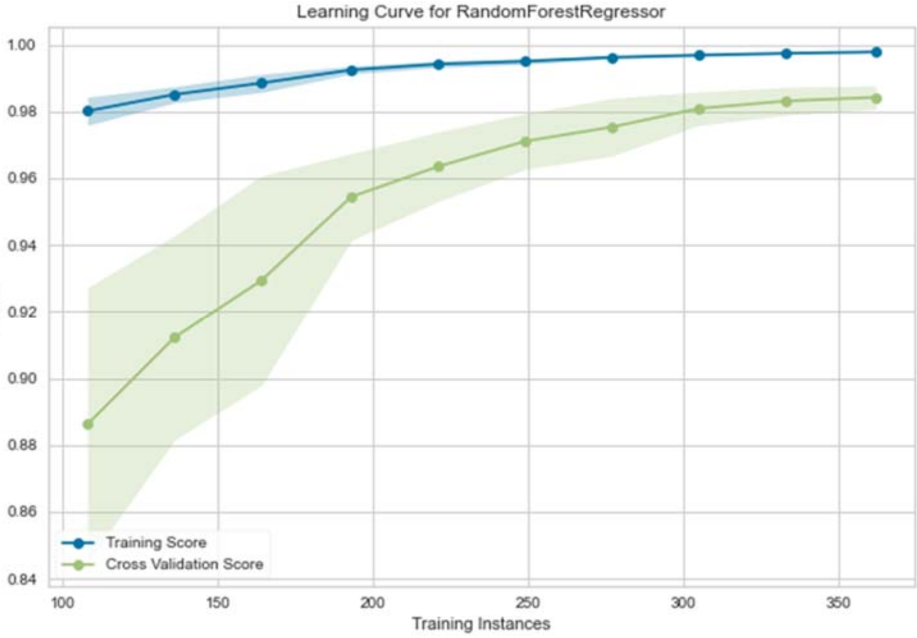

Learning Curve for RandomForestRegressor
1.00
0.98
0.96
0.94
0.92
0.90
0.88
0.86
0.84
Score
Training Score
Cross Validation Score
100
150
200
250
300
350
Training Instances

d

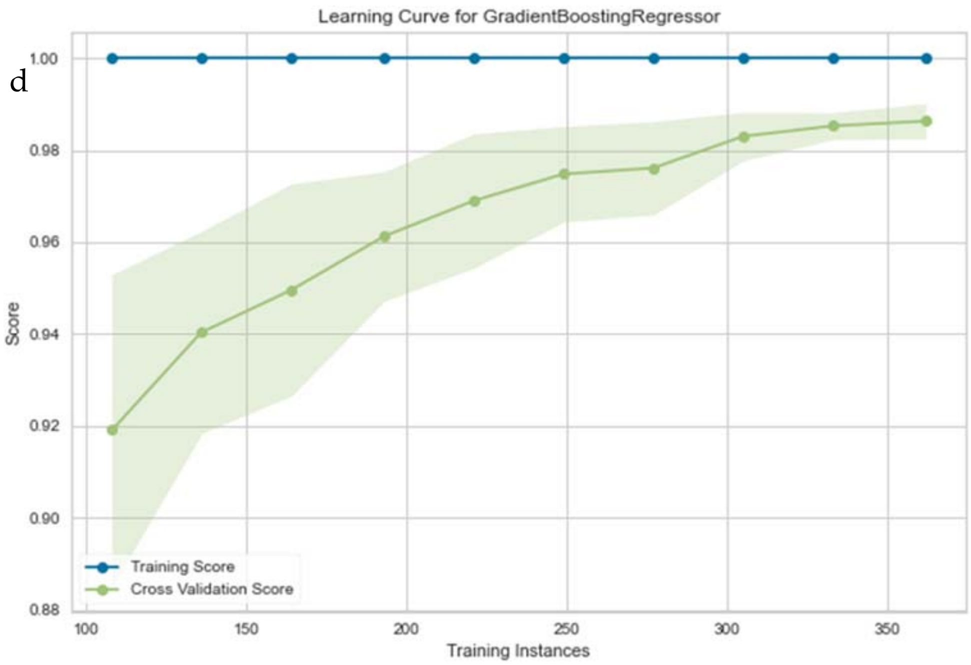

Figure S2. Different machine learning models' learning curve for a) Decision tree regressor, b) KNN regressor, c) Random forest regressor, and d) Gradient boosting regressor.
